# Development of a Test of Suprathreshold Acuity in Noise in Brazilian Portuguese: A New Method for Hearing Screening and Surveillance

**DOI:** 10.1155/2014/652838

**Published:** 2014-08-28

**Authors:** Nara Vaez, Liliane Desgualdo-Pereira, Alessia Paglialonga

**Affiliations:** ^1^São Paulo School of Medicine, Federal University of São Paulo (EPM-UNIFESP), Rua Botucatu 802, 04023-900 São Paulo, SP, Brazil; ^2^National Research Council of Italy (CNR), Institute of Electronics, Computer and Telecommunication Engineering (IEIIT), Piazza Leonardo da Vinci 32, I-20133 Milano, Italy

## Abstract

This paper describes the development of a speech-in-noise test for hearing screening and surveillance in Brazilian Portuguese based on the evaluation of suprathreshold acuity performances. The SUN test (Speech Understanding in Noise) consists of a list of intervocalic consonants in noise presented in a multiple-choice paradigm by means of a touch screen. The test provides one out of three possible results: “a hearing check is recommended” (*red* light), “a hearing check would be advisable” (*yellow* light), and “no hearing difficulties” (*green* light) (Paglialonga et al., Comput. Biol. Med. 2014). This novel test was developed in a population of 30 normal hearing young adults and 101 adults with varying degrees of hearing impairment and handicap, including normal hearing. The test had 84% sensitivity and 76% specificity compared to conventional pure-tone screening and 83% sensitivity and 86% specificity to detect disabling hearing impairment. The test outcomes were in line with the degree of self-perceived hearing handicap. The results found here paralleled those reported in the literature for the SUN test and for conventional speech-in-noise measures. This study showed that the proposed test might be a viable method to identify individuals with hearing problems to be referred to further audiological assessment and intervention.

## 1. Introduction

Age-related hearing loss and noise-induced hearing loss are common health problems in adults and older adults. The World Health Organization [1, 2] recently indicated hearing loss as the first among the twenty leading causes of moderate-to-severe disability in the adult population. It is estimated that 46% of persons over the age of 60 years, and as many as 83% over 70 years, experience some degree of hearing loss [3]. Epidemiological data suggest that nearly 1 in 25 older adults develops a hearing impairment each year [4] and that almost one in two persons with hearing impairment shows a decline in their hearing over a 5-year period [5]. Yet, despite the high prevalence and incidence, and notwithstanding the significant effects on speech communication, social participation, personal well-being, quality of life, and cognitive decline [6–8], hearing loss in adults is still largely underdetected and undertreated [9, 10]. The impact of hearing problems in adults can be substantial because many people may get used to the slow progression of their impairment and typically do not seek help or seek help very late, on average from eight to ten years after the onset of first complaints and symptoms, once the associated handicap and disability have a severe impact on their lives [10–12]. This brings along considerable burden on the health care system and society as the consequences of untreated hearing loss are huge not only in older adults but also, and increasingly, in middle-aged adults who are still fully engaged in their working life as well as in their social and personal domains [6, 11–13].

Recently, some initiatives and a number of studies have promoted the definition and implementation of effective programs of hearing screening, prevention, surveillance, and care for adults and older adults, particularly in Europe and in the United States [10, 14–16], although the value and need for community hearing screening in adults are still controversial. As reported by the US Preventive Services Task Force reports, current evidence is insufficient to assess the balance of benefits and harms of screening in adults without symptoms of hearing loss, and additional research is needed to understand the effects of screening for hearing loss compared with no screening on health outcomes [17, 18]. Nevertheless, the research reported by Yueh et al. in 2010 [19] did indeed suggest that hearing screening can be a catalyst in moving individuals from just a hearing loss to actually doing something about it. Moreover, if the outcome of the screening is normal, then it still might provide the individual with an increased awareness of the potential problem and could allow the audiologist to provide people who underestimate their hearing problems with information about the condition, stimulating their motivation and attitude to seek help [20]. Awareness could lead to employment of preventative measures or earlier detection of a hearing problem, before the onset of hearing handicap and disability [16, 21, 22] so that health care can be delivered in a timely manner. Early intervention is particularly important, also in view of recent evidence showing that age-related hearing loss may be independently associated with poorer cognitive functioning [8, 23] and incident dementia [24, 25] and, also, in view of the hypothesis that rehabilitative therapies and devices may, plausibly, help to limit the cascade of negative effects of hearing loss and reduce the risk of cognitive decline [25]. However, the potentially significant benefits of rehabilitation on the cognitive domain were investigated and demonstrated by only one randomized controlled trial up to now [26].

Unfortunately, there is still lack of large scale hearing screening and surveillance programs in many developed and developing countries, including Brazil, even though some recent studies have demonstrated that the implementation of adult hearing screening might be a cost-effective way to reduce unmet need for hearing aids and improve quality of life among older adults [16, 27]. The availability of reliable methods to identify hearing problems is a crucial prerequisite for the implementation of effective screening, prevention, and surveillance programs. In the past ten years, some novel techniques have been specifically developed for hearing screening and surveillance, particularly in adults and older adults. Relevant examples are digits-based tests for remote testing (at-home self-screening) by telephone or Internet [28, 29], adaptive tests based on multiple-choice discrimination of logatomes [30, 31], and tests of suprathreshold acuity in noise such as the SUN (Speech Understanding in Noise) test [32–35]. The SUN test is an automated, user-operated speech-in-noise test which is made up of short list of intervocalic consonants (VCVs, vowel-consonant-vowel, e.g.,* afa, aja*,…) that are presented in a three-alternative forced choice (3AFC) paradigm by means of a touch sensitive screen. Stimuli are delivered monaurally with unmodulated background speech-shaped noise, which is adequately adapted in level for each stimulus. The test score is computed as the number of VCVs correctly identified and, based on this score, the test outcome is given in a self-explanatory way: either “no hearing difficulties” (*green* light), “a hearing check would be advisable” (*yellow* light), or “a hearing check is recommended” (*red* light). Previous studies have shown that this test is a fast, reliable, easy to use tool that proved to be viable to identify early hearing difficulties in clinical as well as nonclinical settings, including unchecked ambient noise (up to 65 dBA) [32, 33].

The aim of this study was to develop a test of suprathreshold acuity in noise, the SUN test, in the Brazilian Portuguese language. This was done through three main steps, that is, (1) the development of the “building blocks” of the test (i.e., speech stimuli and background noise); (2) the definition of the test sequence (i.e., the list of stimuli and the associated levels of background noise); and (3) the optimization of the test outcomes so that the agreement between the test outcomes and hearing impairment was the highest. The development procedure and the most relevant results, as well as the overall findings in a population of 101 subjects, will be described in the following sections.

## 2. Materials and Methods

### 2.1. Development of the “Building Blocks”: Speech Stimuli and Background Noise

Sixteen VCVs were recorded for the Brazilian Portuguese language (*aba, aca, aça, acha, ada, afa, aga, aja, ala, alha, apa, ara, arra, ata, ava, *and* aza*). Of the 19 consonants in the Brazilian alphabet, three (i.e., /m/, /n/, and/*η*/) were not used because the phonemes* ama*,* ana,* and* a*η*a* have a nasal sound which is reflected in the nasalization of the first vowel [36] and would give a cue for discrimination, introducing a bias in the test results. Following the same procedure as in the other language versions of the SUN test [32], VCV utterances were recorded as single exemplars in a sound-treated room by a professional, native-Brazilian, male speaker who was instructed to pronounce the VCVs with no prosodic accent, with the stress on the first vowel and with constant pitch across the list. Stimuli were recorded in a professional recording studio using a Neumann TLM 103 microphone, a SSL S4000 64-channels mixer, Motu HD 192 A/D converters (44.1 kHz, 16 bit), and GENELEC 1025A control room monitor. The level of VCV recordings was digitally equalized across the set to meet the “equal speech level” requirement as set in the ISO 8253-3:1996(E) Standard for Speech Audiometry [37]. The background noise in the test was a steady speech-shaped noise, that is, a white noise filtered with the long-term average speech spectrum of the Brazilian Portuguese language by a male speaker. To deliver the test in the 3AFC multiple-choice task, each VCV was combined with two wrong alternatives. This was done according to the “maximal opposition” criterion: the two wrong alternatives differed from the spoken stimulus in all the relevant feature dimensions: voicing, manner, and place of articulation (e.g.,* ata*,* alha*, and* ava*) [32].

### 2.2. Definition of the Test Sequence

The definition of the test sequence entailed the optimization of the list of stimuli and the associated levels of background noise. The rationale was to set the level of noise for each VCV so that the intelligibility of stimuli was approximately the same across the test list (for details, see [32]). To equalize the intelligibility of stimuli, the presentation SNR (signal-to-noise ratio) for each VCV was set above the reception threshold (RT), which is theoretically defined as the SNR where the psychometric curve reaches 79.4% correct responses in a 3AFC task [38].

To estimate the RTs, the psychometric curves of the 16 VCVs were measured for a wide range of SNRs, from chance performance to 100% recognition, that is, from −10 dB to +6 dB SNR, in 2 dB steps. In this experiment, the output level of VCVs was fixed at 60 dB HL (i.e., above 100% correct responses in normal hearing subjects [39]) and the output level of noise was varied, from 70 dB HL to 52 dB HL, to set the desired SNR. Noise onset and offset were 500 ms before and 100 ms after the VCV onset and offset, respectively. To familiarize participants with the test stimuli and procedure, VCVs were also presented at the beginning of the test sequence at a comfortable presentation SNR of +8 dB (above 100% recognition [40, 41]). Stimuli were presented monaurally through headphones in the right ear and in the left ear, in two separate sessions. At each SNR, stimuli were presented twice, resulting in a total of 320 stimuli presented per ear (16 VCVs × 10 SNRs × 2 repeated presentations). The resulting test sequence was completely randomized across SNRs and across VCVs to limit adaptation to noise levels and learning.

Participants were 30 young adults (15 males, 15 females; age range: 20–25 years; mean: 22.5 years; s.d. 1.89), otologically normal as in the ISO 7029:2000 Standard [42].  Table 1 (upper part) summarizes the pure-tone detection thresholds measured in these subjects.

### 2.3. Optimization of the Test Outcomes

The optimization of the test outcomes was performed by setting two cut-off scores (*T*_1_ and *T*_2_) between the three categories of test results (“no hearing difficulties”: score ≥ *T*_1_; “a hearing check would be advisable”: *T*_2_ < score < *T*_1_; and “a hearing check is recommended”: score ≤ *T*_2_) so that the agreement with the reference pure-tone benchmarks for pure-tone screening was the highest (for details, see [32]). On a monaural basis, the outcomes of pure-tone screening in each tested ear were defined by three different classes and the percentage of correct classifications, as well as the sensitivity and specificity of the test, were measured as a function of the different values of *T*_1_ and *T*_2_ to find the cut-off scores that maximised them. The three classes were:

 Class I (pure-tone hearing thresholds ≤ 40 dB HL at 1, 2, and 4 kHz), that is, ears that would pass a pure-tone screening at a level of 40 dB HL (a cut-off threshold of 40 dB HL was used as this reference value is recommended for pure-tone screening in nonclinical settings and, in general, where the ambient noise is not controlled [43], as well as for screening adults and older adults [9, 44, 45]).
 Class II (pure-tone hearing thresholds > 40 dB HL at 4 kHz and ≤ 40 dB HL at 1 and 2 kHz), that is, ears that might either fail or pass a pure-tone screening at 40 dB HL, depending on whether the frequency 4 kHz is included or not in the pass/refer criterion, respectively [43, 44].
 Class III (pure-tone hearing thresholds > 40 dB HL at 1 or 2 kHz), that is, ears that would fail a pure-tone screening, irrespective of the criterion for high-frequency hearing thresholds [43–45].

On a binaural basis, the World Health Organization (WHO) [46] criterion for disabling hearing impairment was considered the reference benchmark (i.e., hearing threshold level, averaged over 0.5, 1, 2, and 4 kHz, >40 dB HL in the better ear; hearing aids are usually recommended). The SUN test results in the two ears were combined in a way that a binaural “fail” was given when the subject received “a hearing check is recommended” (*red* light) in at least one ear, as defined in [32]. The sensitivity and specificity of the test to identify disabling hearing impairment were measured.

Moreover, to evaluate the agreement between the test outcomes and self-perceived hearing handicap, subjects were also asked to fill in the HHIE-S (hearing handicap for the elderly screening) questionnaire [44], in the Brazilian Portuguese version [47]. The HHIE-S is a 10-item questionnaire that scores the emotional and social consequences of hearing impairment on a scale of 0 to 40 and, based on this score, classifies subjects into three categories of hearing handicap: “no handicap” (score ≤ 8), “mild-to-moderate handicap” (score in the range of 10–22), or “significant handicap” (score ≥ 24).

Participants were 101 native-Brazilian adults and older adults (43 males, 58 females; age range: 26–85 years; mean 52.7 years; s.d. 15.15) recruited in order to have a wide range of pure-tone thresholds represented across the study sample, from normal hearing to severe hearing loss. The maxima, mean values, and standard deviation (s.d.) of pure-tone thresholds measured in this group of subjects are reported in Table 1 (lower part). The SUN test and pure-tone audiometry were administered in both ears, with only one exception, for a total of 201 ears tested.

### 2.4. Equipment

A PC connected to the RCA analogue input of a clinical audiometer (Auditec PAC-200 with TDH-39 headphones) was used to deliver the stimuli. The speech output was calibrated by using a 1 kHz tone as in the “equal speech level” requirement in the ISO 8253-1:1996 Standard for speech audiometry [48]. A touch sensitive screen (resistive LCD Viper 10.4′′; resolution: 800 × 600 pixels; brightness: 350 cd/m2; contrast ratio: 250 : 1) was used to display the written alternatives, record the subjects' responses, and display the test score. The software and user interface for experiments were implemented in MATLAB (R2007b, v. 7.5.0.342, MathWorks). Testing was carried out in low ambient noise (as in the ISO 8253 1:1989 Standard for Pure Tone Audiometry [49]). The experiments were conducted in accordance with the Declaration of Helsinki and were approved by the Ethics Committee for Research at UNIFESP (Comitê de Ética e Pesquisa da Universidade Federal de Sao Paulo, Plataforma Brasil). Participants were informed of the study framework and procedures before testing and agreed to the use of their clinical data for research purposes.

## 3. Results and Discussions

**Figure 1:**
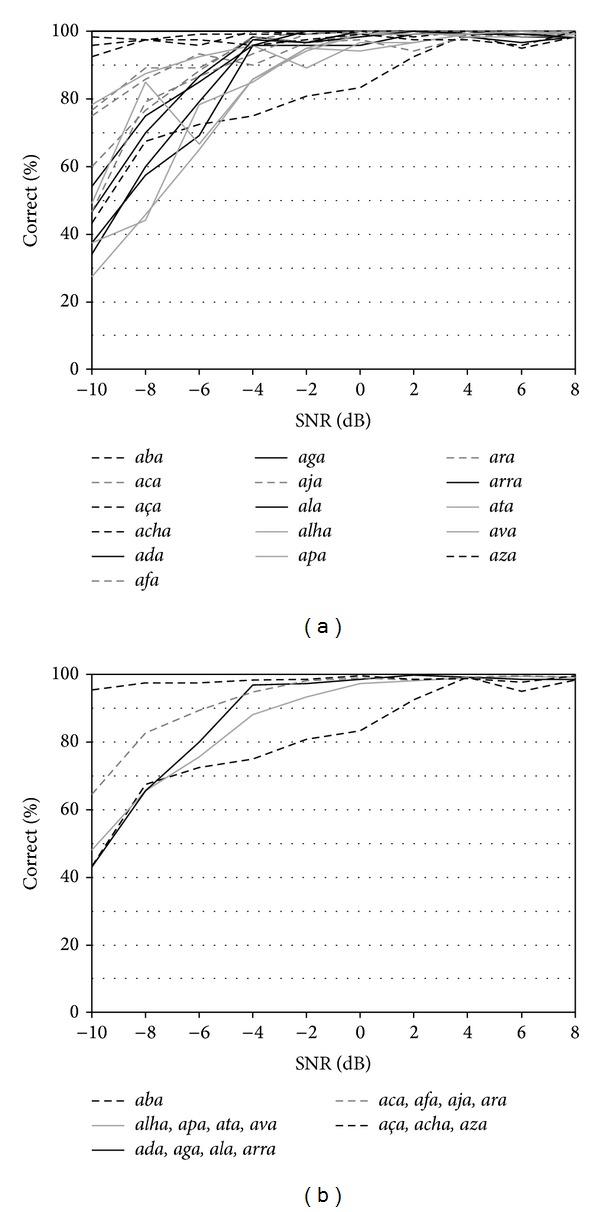
(a) Psychometric curves of the sixteen spoken VCVs as a function of the SNR, from −10 dB to +8 dB, in 2 dB steps. (b) Psychometric curves of the five subsets of VCVs as a function of the SNR.

Figure 1(a) shows the psychometric curves of the 16 VCVs as a function of the SNR, from −10 dB to +8 dB. A wide range of* RTs* was observed across the set, from values lower than 10 dB (e.g.,* aza*,* acha*) to about +2 dB (e.g.,* aba*), further supporting the need to equalize the intelligibility of test stimuli. Following the analysis of psychometric curves as well as the statistical distribution of results in this group, the presentation SNR was set at +4 dB for the stimulus* aba*, at 0 dB for stimuli* alha*,* apa*,* ata*, and* ava*, at −2 dB for* ada*,* aga*,* ala*, and* arra*, at −4 dB for* aca*,* afa*,* aja*, and* ara*, and at −6 dB for* aça*,* acha*, and* aza*. The average psychometric curves of the five subsets of VCVs are shown in Figure 1(b).

As a result, the SUN test sequence was obtained. It consisted in the set of 16 VCVs, presented in five different subsets at the five presentation SNRs listed above, from the highest (+4 dB) to the lowest SNR (−6 dB). In addition, to help subject familiarize with the test procedure, a short list of VCVs is also presented (but not scored) before the actual test list at a comfortable presentation SNR of +8 dB [32].

**Figure 2:**
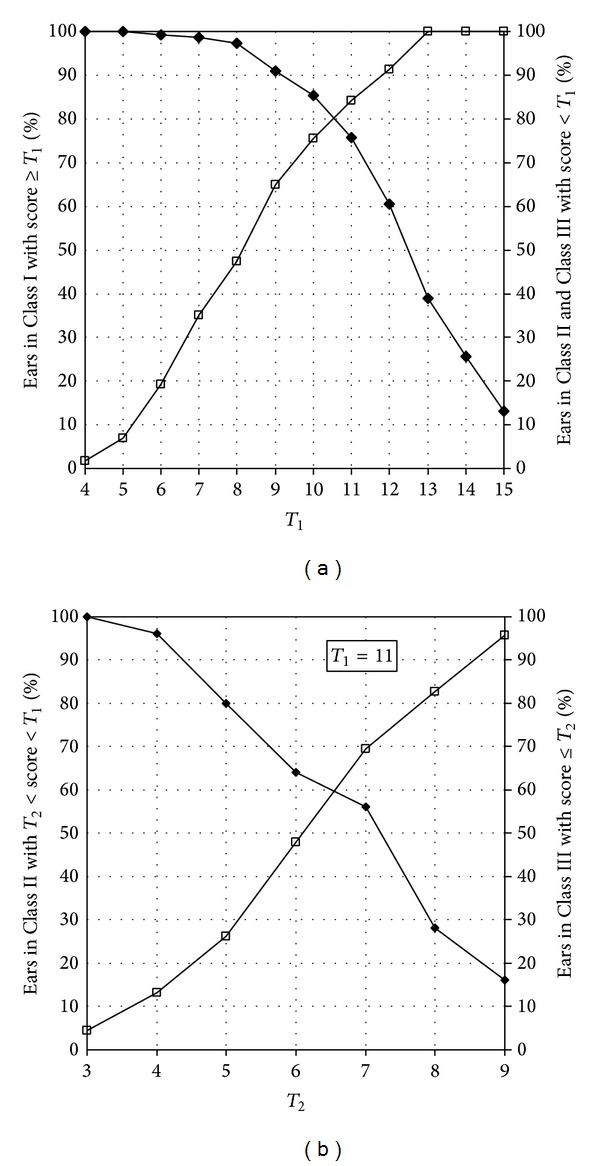
(a) Percentage of ears in Class I with score ≥ *T*_1_ (black marks) and percentage of ears in Class II or Class III with score < *T*_1_ (white marks) when *T*_1_ was systematically varied from 4 to 15. (b) Percentage of ears in Class III with score ≤ *T*_2_ (white marks) and percentage of ears in Class II with *T*_2_ < score < *T*_1_ (black marks) when  *T*_2_  was systematically varied from 3 to 9, and *T*_1_ was set at 11 (as this value provided the best results, as shown in (a)).

Figure 2 shows the percentage of ears in Classes I, II, and III that were classified into the three categories of test outcomes as a function of the two cut-off scores, *T*_1_ (Figure 2(a)) and *T*_2_ (Figure 2(b)). Figure 2(a) shows the percentage of ears in Class I (i.e., ears that would pass a pure-tone screening) with score ≥*T*_1_ (black marks) and the percentage of ears in Class II or Class III (i.e., ears that would not pass a pure-tone screening) with score < *T*_1_ (white marks) for the different values of *T*_1_. The highest agreement between test results and pure-tone screening results was observed with *T*_1_ = 11, which provided a sensitivity of 84% and specificity of 76% to identify ears that might fail a pure-tone screening (Classes II and III). Figure 2(b) shows, the percentage of ears in Class III with score ≤*T*_2_ (white marks) and the percentage of ears in Class II with *T*_2_ < score < *T*_1_ (black marks) as a function of *T*_2_, with *T*_1_ set at 11. Accordingly, the cut-off score *T*_2_ was set at 7 as this provided about 69.6% and 56% correct subclassification between Class II and Class III and a sensitivity of 62% and specificity of 92% to identify ears in Class III. On a binaural basis, the sensitivity and specificity of the SUN test to identify people with disabling hearing impairment, as defined by the WHO criterion [46], were 83% and 86%, respectively, paralleling results reported by previous investigations (e.g., the sensitivity and specificity were 83.8% and 83.9% for the Italian version of the SUN test [32] and 84% and 75% for the English version [33]).

**Figure 3:**
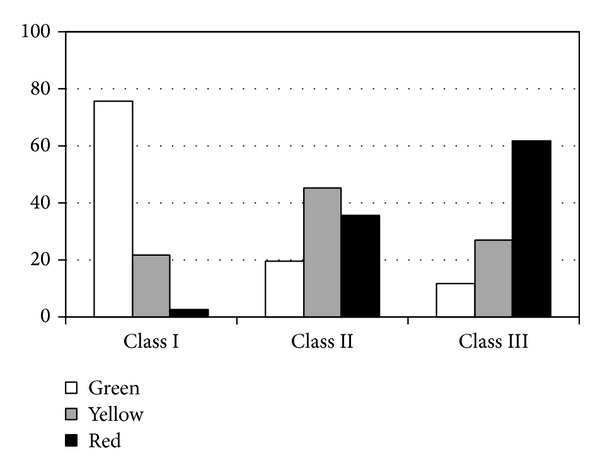
Distribution of the three test outcomes (*green*: “no hearing difficulties”;* yellow*: “a hearing check would be advisable”; and* red*: “a hearing check is recommended”) in ears classified into the three classes of hearing impairment (Classes I, II, and III).

Figure 3 shows, on a monaural basis, the overall distribution of the three test outcomes in ears classified into the three classes of hearing impairment. Of ears in Class I, 75.7% were classified into the* green* category and only a minor percentage (i.e., 2.8%) were classified as* red*. Vice versa, of ears in Class III, an overall percentage of 88.5% were classified as* yellow* (26.9%) or* red* (61.6%). As to ears in Class II (i.e., ears with pure-tone thresholds higher than 40 dB HL at 4 kHz but* not* at frequencies ≤ 2 kHz), the distribution of results was as follows: 19.3%* green*, 45.2%* yellow*, and 35.5%* red*. This outcome might be explained by the fact that puretone thresholds at 4 kHz are typically not* per se* adequate predictors of speech reception threshold, unless they are combined with thresholds at lower frequencies, at 2 kHz or lower [50]. As a matter of fact, Ventry and Weinstein [44] had reported that relying on a fail with pure-tone thresholds at 4 kHz to screen for hearing handicap produces a high refer rate and fails a high proportion of those who report no handicap. Stated differently, adults with high-frequency hearing loss may—or may not—experience difficulties in speech understanding, as reflected by our results. Conversely, when the hearing loss involves frequencies below 4 kHz (as for ears in Class III), the ability to understand speech, particularly in less than ideal listening conditions, significantly decreases because the mid-to-low frequency regions contribute more to speech understanding than high-frequency regions [51–53]. Accordingly, as shown in Figure 3, the agreement between speech understanding (as measured by the proposed test) and pure-tone thresholds is much higher, as expected, for ears in Class I or Class III (i.e., where pure-tone thresholds at frequencies ≤ 2 kHz were lower or higher than 40 dB, resp.) than for ears in Class II.

It might be observed that a mismatch between results of tests of speech recognition in noise and tests of pure-tone detection in quiet is largely justified, particularly in adults and older adults [51, 54, 55]. For example, in line with this, our results showed that 2.8% of ears in Class I were in the* red* category, which may be explained by possible early hearing difficulties that are not significant yet and are not detected by hearing threshold testing. Vice versa, 11.5% of ears in Class III were in the* green* category, possibly because of gradual adaptation to speech-in-noise recognition in people with hearing difficulties and, also, because of the 33% guess rate in the 3AFC task which may lead to higher-than-expected scores. Noticeably, the results found here are in line with findings reported in the literature for other speech-in-noise tests (sentence, word, or nonsense syllable tests) that are typically used for hearing assessment in the clinic [50, 56–59], overall confirming that the SUN test can estimate speech recognition performance reliably and quickly in adults and older adults. In general, the agreement between speech-in-noise testing and pure-tone testing widely varies as it depends on the speech material, noise, and protocols that are used, but typically the correlation between speech recognition and pure-tone thresholds, particularly for consonants and short phonemes, is below 0.7 [58, 59].

**Figure 4:**
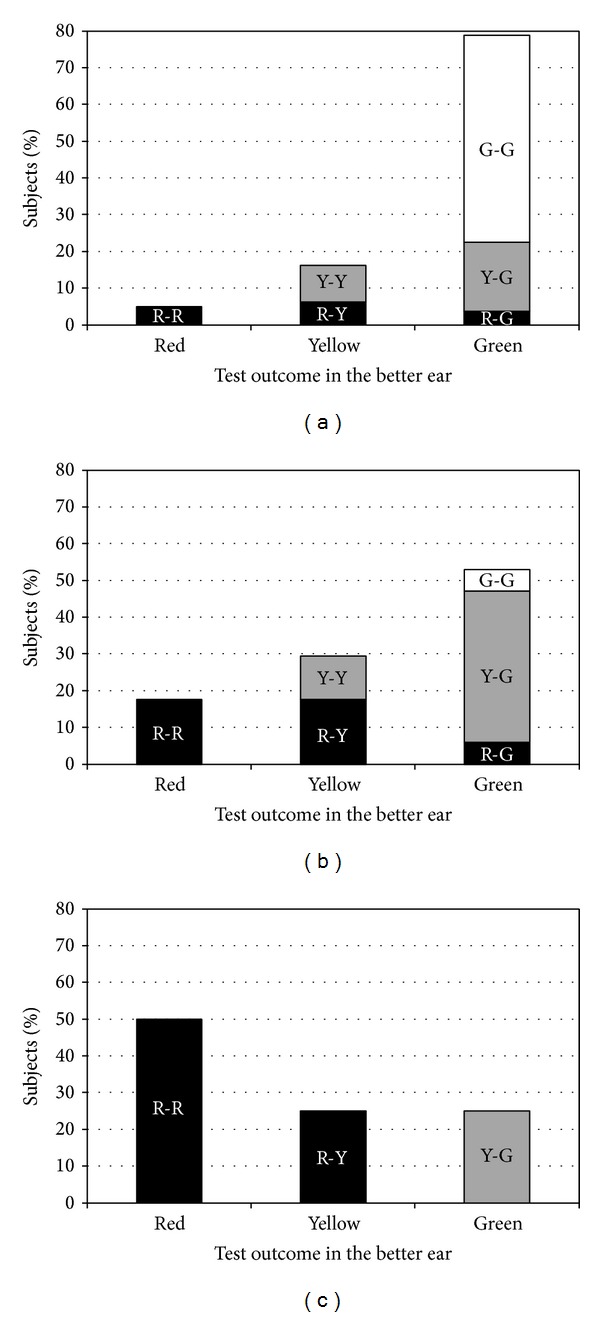
Distribution of the three test outcomes in subjects classified into the three categories of hearing handicap, as measured by the HHIE-S questionnaire: (a) “no handicap” (*N* = 80); (b) “mild-to-moderate handicap” (*N* = 17); (c) “significant handicap” (*N* = 4). For each category of hearing handicap, subjects were divided into three subgroups according to the test results in their better ear, that is,* red* (left-hand bar),* yellow* (center bar), and* green* (right-hand bar). Each of these bars also shows the distribution of scores obtained by those subjects in the other ear:* red* (black bars),* yellow* (grey bars), or* green* (white bars).

Figure 4 shows the distribution of the three SUN test outcomes in subjects classified into the three categories of hearing handicap, as measured by the HHIE-S questionnaire: “no handicap” (Figure 4(a)), “mild-to-moderate handicap” (Figure 4(b)), and “significant handicap” (Figure 4(c)). In the group of subjects with no handicap (*N* = 80, Figure 4(a)), an overall percentage of 78.8% had* green* in their better ear (right-hand bar); of these, 56.3% had* green* in both ears, whereas 18.7% and 3.8% had* yellow* or* red* in the other ear, respectively. In this group, a minor percentage of subjects (i.e., 5%, left-hand bar) obtained* red* in both ears, whereas about 16% of subjects (center bar) obtained* yellow* either in one ear (6.3%) or in two ears (10%). These data suggest that, in individuals with no self-perceived hearing handicap, the better ear plays a major role as most subjects (78.8%) had very good outcomes (*green* light) in at least one ear. As a matter of fact, good hearing functioning, at least in one ear, is likely to help them to cope with daily (binaural) listening situations, thus lowering the degree of self-perceived hearing handicap. In the group with mild-to-moderate handicap (*N* = 17, Figure 4(b)), the percentage of subjects who obtained a* red* outcome in both ears increased from 5% to 17.6% (left-hand bar) and the percentage of those who had* green* in at least one ear decreased from 78.8% to 52.9% (right-hand bar), with a parallel decrease in the percentage of those who had* green* in both ears, from 56.3% (45/80) to 5.9% (1/17). The percentage of subjects who obtained* yellow* in their better ear was 29.5% (17.7% had* red* in one ear and 11.8% had* yellow* in the other ear). The distribution of test results in this group indicates that a higher proportion of subjects with mild-to-moderate handicap are likely to have poorer speech recognition in noise, as measured by the SUN test, in both ears (left and center bars: 47.1%). In the small group of subjects who reported significant handicap (*N* = 4, Figure 4(c)), two (50%, left-hand bar) had* red* in both ears, one (25%, center bar) obtained* yellow* in one ear and* red* in the other ear, and one (25%, right-hand bar) had* yellow* in one ear and* green* in the other ear. In summary, the percentage of subjects who obtained* red* outcomes in both ears markedly increased with increasing self-perceived handicap (from 5% to 17.6% and 50%); vice versa, the percentage of subjects who obtained* green* in at least one ear markedly decreased with increasing handicap (from 78.8% to 52.9% and 25%—this last percentage corresponding to one out of four subjects). Despite the very small sample size, results in Figure 4(c) confirm the tendency observed in Figure 4(b): subjects with significant hearing handicap tend to have poor speech recognition in both ears. Additional research in a wider sample of individuals with significant handicap is needed to further support these findings and to fully understand the role of the better (leading) ear in limiting the degree of self-perceived hearing handicap. Overall, these results indicate that the test outcomes were in line with the degree of self-perceived hearing handicap, as measured by the HHIE-S.

## 4. Conclusions

This paper described the development of a test of suprathreshold acuity in noise in the Brazilian Portuguese language, that is, the SUN (Speech Understanding in Noise) test, a user-operated speech-in-noise test for hearing screening and surveillance, already available in the English, Italian, German, and French languages. This was done by (1) developing its building blocks (speech stimuli and noise); (2) defining the list of stimuli and the associated levels of background noise; and (3) optimizing the test outcomes in a population of adults and older adults with varying degrees of hearing impairment and hearing handicap, including normal hearing. The results obtained with the proposed test fully paralleled those obtained in previous studies in the other language versions [32–35] and were in line with results reported for conventional clinical tests of speech-in-noise recognition [50, 56–59]. Evidence from a group of 101 subjects indicated that the present test can be a viable measure for screening or surveillance programs to identify individuals with hearing impairment and hearing handicap to be referred to further audiological assessment and intervention.

It is recognized that this is a preliminary investigation on a relatively small study sample and that more studies and research need to be conducted on this topic, on a bigger group of subjects. For example, it would be interesting to investigate the association between the different degrees and types of hearing loss (including conductive hearing loss) and the test results on a large representative sample or, also, to measure the correlation with conventional speech-in-noise tests or other clinical assessment measures, including questionnaires different than the HHIE-S. Moreover, additional research is still needed to further evaluate the performance of the proposed test and to fully assess its feasibility as a screening tool, also taking into account the peculiar characteristics of the different populations and countries (either developed or developing). Future studies need to be performed in larger samples of subjects to fully assess the viability of the test for the general population; particularly, it would be important to promote large screening campaigns in Brazil by using the test developed here, also searching for specific solutions to provide accessible and efficient services to help people in remote or underserved areas. The importance of these studies would cross the borders of this specific country as valuable, practical indications would be set that might be useful to the scientific and clinical community at large for the future definition and implementation of successful early detection and intervention services for adults.

## Figures and Tables

**Table 1:** Maxima, mean values, and standard deviation (s.d.) of pure-tone thresholds measured in the group of 30 normal hearing young adults (NHY) and in the group of 101 adults with varying degrees of hearing impairment and handicap (ADU).

	Right ear					Left ear				
Frequency (kHz)	0.5	1	2	4	8	0.5	1	2	4	8
NHY										
Max (dB HL)	15	15	10	15	15	20	15	10	15	10
Mean (dB HL)	6.7	4.3	2.3	2.8	2.0	7.5	4.3	2.2	2.3	1.3
S.d.	6.61	5.53	3.88	4.29	3.62	6.79	4.87	3.39	4.30	2.60
ADU										
Max (dB HL)	50	60	75	100	85	65	60	70	85	95
Mean (dB HL)	20.0	18.0	19.6	27.3	32.7	19.4	17.2	18.7	27.4	32.8
S.d.	10.38	13.84	17.91	23.71	26.66	10.68	13.46	17.63	22.80	27.98
